# The Efficacy and Safety of Imusil® Tablets in the Treatment of Adult Patients With Mild COVID-19: A Prospective, Randomized, Multicenter, Open-Label Study

**DOI:** 10.7759/cureus.35881

**Published:** 2023-03-07

**Authors:** Mahesh Abhyankar, Dilip Kadam, P. Raghavendra Reddy, Mohammed Zaki Siddiqui, M Ratheesh, Tariq Jagmag, Jayesh Tilwani

**Affiliations:** 1 Internal Medicine, Glowderma Lab Private Limited, Mumbai, IND; 2 Internal Medicine, Care Multispeciality Hospital, Pune, IND; 3 Internal Medicine, Renova Neelima Hospital, Hyderabad, IND; 4 Internal Medicine, Maharani Laxmi Bai Medical College, Jhansi, IND; 5 Biochemistry, St. Thomas College, Kottayam, IND; 6 Medical Affairs, Glowderma Lab Private Limited, Mumbai, IND

**Keywords:** interleukin 6, rt-pcr, safe, d-dimer, crp

## Abstract

Introduction

Coronavirus disease 2019 (COVID-19) is a serious concern of the new era. Along with antiviral synthetic medications, there is a need to discover efficacious herbal antiviral medicines with minimum side effects in patients against COVID-19. This study aimed to assess the efficacy and safety of Imusil® among patients with mild COVID-19.

Methods

A prospective, randomized, multicenter, open-label, interventional study was conducted in patients with mild COVID-19 infection. Patients received either Imusil one tablet four times a day (seven days) along with the standard of care (SoC) or only SoC. The study endpoints were reverse transcription-polymerase chain reaction (RT-PCR) negativity, changes in cycle threshold (CT), clinical improvement, change in blood inflammatory indexes, and safety assessment.

Results

A total of 100 patients were enrolled, and 98 received at least one dose of treatment. The median age of patients was 36.0 years, and 58 were males. By day 4, 85.4% of patients in the Imusil+SoC group tested negative for RT-PCR compared to 64% of patients exhibiting the same outcome in the SoC group (P=0.0156). After eight days, clinical improvement was observed in all patients from the Imusil+SoC group, while in the SoC group, clinical improvement was observed in 94.0% of patients (P=0.4947). During follow-up visits, the average C-reactive protein (CRP) levels decreased from baseline in both treatment groups. The decrease in the levels of CRP (-7.3 mg/dL versus -5.5 mg/dL), D-dimer (-231.0 ng/mL versus -151.6 ng/mL), and interleukin 6 (IL-6) (-2.3 pg/mL versus -2.0 pg/mL) at eight days was comparatively higher in the Imusil+SoC group versus the SoC group. There were no serious treatment-emergent adverse events in the drug arm.

Conclusion

Imusil provides effective antiviral activity and safety in mild COVID-19 patients. Imusil ensures faster RT-PCR negativity and clinical improvement and ensures effective reduction of inflammatory markers such as CRP, D-dimer and interleukin 6.

## Introduction

The global pandemic coronavirus disease 2019 (COVID-19) is a serious concern of the new era that comprises a broad spectrum of severity, ranging from asymptomatic to severe. Severe cases of COVID-19 can be complicated by acute respiratory distress syndrome and lung failure leading to death [1]. Cytokine storm syndrome leading to hyper-inflammation is associated with poor prognosis in COVID-19. Evidence suggests that elevated levels of pro-inflammatory cytokine interleukin 6 (IL-6) were associated with the disease severity of COVID-19 [2,3]. The infection induces pathological responses, including increased inflammation, oxidative stress, and apoptosis [4].

Many interventions and therapeutics have been proposed to combat the viral infection-induced inflammation and oxidative stress contributing to the etiology and pathogenesis of COVID-19. However, these methods have not significantly improved treatment outcomes [4]. Antiviral synthetic medications such as favipiravir and remdesivir are initially effective for the treatment of COVID-19 infection [5,6]. Nevertheless, they are linked with several adverse effects that are harmful to patients with heart disease, diabetes, and kidney disease [7-9]. Therefore, the need for inherently safe biologically active therapeutics of plant origin is required for reducing and managing the risk of viral infections including COVID-19. Traditional medicines and their combination had shown to result in the recovery of patients with COVID-19 [10]. The Ministry of Ayurveda, Yoga and Naturopathy, Unani, Siddha and Homeopathy (AYUSH), India, has identified neem, kutki (*Picrorhiza kurroa*), guduchi (*Tinospora cordifolia*), amla (*Emblica officinalis*), tulsi (*Ocimum sanctum*), ashwagandha (*Withania somnifera*), ginger (*Zingiber officinale*), turmeric (*Curcuma longa*), licorice (*Glycyrrhiza glabra*), aloe, shatavari (*Asparagus racemosus*), almond, cinnamon, black pepper, drumstick, and broccoli as important first-level ayurvedic herbs, which play a crucial role in the defense against this novel viral infection [11].

Imusil® (kutki extract 200 mg+guduchi extract 60 mg+amla extract 60 mg), a novel polyherbal formulation, is being explored in a recently published article for its anti-inflammatory and anti-COVID-19 effect in alleviating the inflammatory responses and thereby improving survival outcomes [12]. Kutki, guduchi, and amla possess immunomodulatory and antioxidant activities. Kutki suppresses the production of reactive oxygen species by inhibiting Nicotinamide adenine dinucleotide phosphate oxidase and pro-inflammatory cytokines. A molecular docking study of guduchi revealed a good activity against the main protease of the virus. Currently, amla is essentially utilized to boost immunity against COVID-19 infection [11].

The previous article elucidated Imusil as a potent anti-inflammatory and antioxidant agent [12]; however, the efficacy and safety of such a combinatorial drug in treating patients with COVID-19 are yet to be explained. Therefore, the present study aimed to evaluate the efficacy and safety of Imusil in mild COVID-19 patients.

## Materials and methods

Study design and setting

This prospective, randomized, multicenter, open-label, interventional study was conducted on patients diagnosed with mild COVID-19 infection visiting three tertiary care centers in India (Jhansi, Hyderabad, and Pune) between 9 March 2022 and 7 May 2022. The study was conducted in accordance with ethical principles that are consistent with the Declaration of Helsinki, the International Conference on Harmonization Good Clinical Practice, and the applicable legislation on noninterventional studies. The study protocol was approved by the institutional ethics committees or review boards of the individual study centers (ECR/1393/Inst/UP/2020, ECR/807/Inst/TG/2016/RR-19, and ECR/1662/Inst/MH/2022). Written informed consent was taken before study participation by all patients.

Inclusion criteria

Adult patients (≥18 years old) of either sex, with confirmed mild COVID-19 infection (World Health Organization {WHO} ordinal score: 2-3), and patients with one or more of the symptoms of fever of ≥100.4°F, cough, sore throat, headache, nasal congestion, malaise, diarrhea, loss of smell, or loss of taste were included in the present study.

Exclusion criteria

Patients with a history of severe infections, including pneumonia and septicemia, or patients who failed to control systemic fungal, bacterial, or viral infection were excluded from the study. Patients with cardiac or pulmonary disease, renal and hepatic impairment (creatine of ≥2 mg/dL, liver enzymes and bilirubin 2.5 times the upper limits of normal (ULN), and alkaline phosphatase 1.5 times ULN), diabetes, obesity, metabolic syndrome, human immunodeficiency virus (HIV) or hepatitis B or C, or neurological or psychiatric disorders were excluded from the study. Patients who received herbal immunosuppressive therapy or other investigational drugs within the previous 30 days of screening or who were hypersensitive to any herbal medication containing kutki, guduchi, or amla extracts were also excluded from the study.

Randomization

The eligible patients were randomized (1:1) in two groups. Group 1 involved treatment with test drug Imusil (kutki extract 200 mg+guduchi extract 60 mg+amla extract 60 mg) tablets plus standard of care (SoC), while group 2 involved treatment with SoC. The SoC was based on the Clinical Management Protocol: COVID-19, Government of India Ministry of Health and Family Welfare Directorate General of Health Services (Electronic Medical Record {EMR} Division). The SoC in mild COVID-19 cases consisted of symptomatic medication, including paracetamol, antitussive and multivitamins, and empiric antimicrobials.

Treatment regimen

Imusil was administered at a dose of one tablet four times daily for seven days. The study involved a screening period (day 1) and a treatment period, which included seven days of dosing. Patients were monitored from the start of treatment. The procedures and assessments were performed during the follow-up period (day 4) and at the end of the study (day 8). Recording of vital signs including saturation of peripheral oxygen (SpO_2_), physical examination, real-time reverse transcription-polymerase chain reaction (RT-PCR) report, detection of inflammatory markers (C-reactive protein {CRP}, D-dimer, and IL-6), clinical assessment based on a WHO ordinal scale (Table 1), and recording of concomitant medications and adverse events (AEs)/serious AEs (SAEs), if any, were done during the study period. Patients were followed for eight days after randomization.

**Table 1:** WHO ordinal scale RRT, renal replacement therapy; ECMO, extracorporeal membrane oxygenation; WHO, World Health Organization

Patient state	Descriptor	Score
Uninfected	No clinical or virological evidence of infection	0
Ambulatory	No limitation of activities	1
	Limitation of activities	2
Hospitalized mild disease	Hospitalized and no oxygen therapy	3
	Oxygen therapy by mask or nasal prongs	4
Hospitalized mild disease	Non-invasive ventilation or high-flow oxygen	5
	Intubation or mechanical ventilation	6
	Ventilation+additional organ support: pressors, RRT, and ECMO	7
Dead	Death	8

Endpoints

The primary endpoints were evaluated on day 4 and day 8 and involved efficacy outcomes such as the rate of severe acute respiratory syndrome coronavirus 2 (SARS-CoV-2) RT-PCR negativity and changes in cycle threshold (CT) value in nasopharyngeal and/or oropharyngeal swab, the proportion of patients with a two-point decrease in a WHO ordinal scale, and changes in blood inflammatory indexes for C-reactive protein, IL-6, and D-dimer. The clinical improvement was assessed using a WHO ordinal scale. The secondary endpoints, such as AEs and SAEs, compared to placebo as an add-on to the SoC, were also evaluated during the study.

Sample size

This study planned to enroll 100 patients with mild COVID-19. Each treatment group was intended to have 50 randomized patients in order to achieve at least 90 evaluable patients (excluding 10% dropout), with about 85% power at the 5% one-sided level of significance for the efficacy endpoint comparison (the proportion of patients getting RT-PCR negative by the end of treatment test plus SoC over SoC).

Definition

The WHO ordinal scale is the WHO ordinal severity scale that has been used to predict mortality and guide trials in COVID-19, which includes the following nine points: 0, no clinical or virological evidence of infection; 1, ambulatory and no activity limitation; 2, ambulatory and activity limitation; 3, hospitalized and no oxygen therapy; 4, hospitalized and oxygen mask or nasal prongs; 5, hospitalized and non-invasive mechanical ventilation (NIMV) or high-flow nasal cannula (HFNC); 6, hospitalized, intubation, and invasive mechanical ventilation (IMV); 7, hospitalized and IMV+additional support such as pressors or extracardiac membranous oxygenation (ECMO); and 8, death [13].

Statistical analysis

Data were analyzed using Statistical Analysis System (SAS) version 9.4 (SAS Institute Inc., Cary, NC). Descriptive statistics were used to describe categorical variables (frequency and percentages) and continuous variables (mean and standard deviation {SD} or median and range {depending on the normality of data}). A comparison of quality between the groups was made using one-way analysis of variance (ANOVA) and Kruskal-Wallis test for parametric and non-parametric variables, respectively. A comparison of quantity between the groups was made using the chi-square test. A paired non-parametric Wilcoxon signed-rank test was used to compare variables pre- and post-treatment. A P<0.05 was considered statistically significant.

## Results

Patient disposition

**Figure 1:**
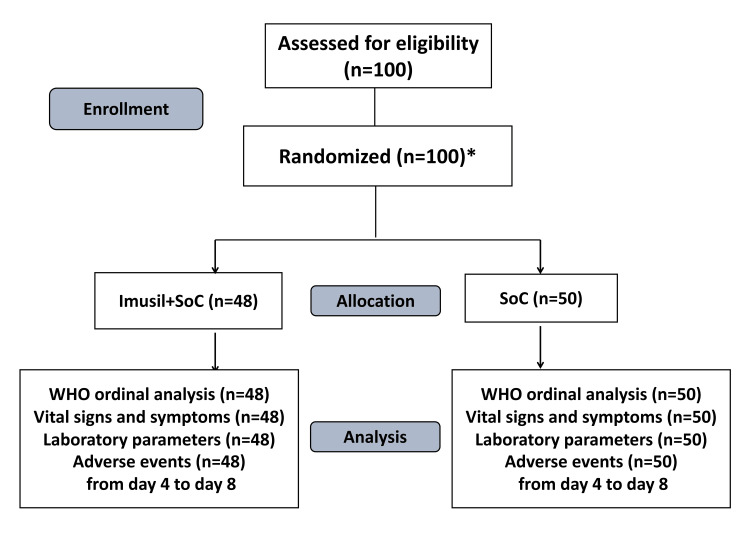
Patient disposition *Two patients who withdrew consent were randomized but did not receive treatment drug SoC, standard of care; WHO, World Health Organization

A total of 100 patients were enrolled, of which two withdrew consent. A total of 98 patients received at least one dose of the investigational product and were included in the safety population (Figure 1).

Baseline characteristics

The median age was 36.0 years, and 58 (59.2%) patients were males. The average body mass index (BMI) of the patient was 23.8 kg/m^2^. The mean height and weight of the patients were 161.5 cm and 61.8 kg, respectively (Table 2).

**Table 2:** Demographic characteristics Data shown as mean (SD), unless otherwise specified SoC, standard of care; SD, standard deviation; BMI, body mass index

Parameters	Imusil+SoC (n=48)	SoC (n=50)	Overall (N=98)
Age (years), median (range)	35.0 (20.0-59.0)	36.5 (18.0-59.0)	36.0 (18.0-59.0)
Sex, n (%)			
Males	17 (35.4)	27 (54.0)	58 (59.2)
Females	31 (64.6)	23 (46.0)	40 (40.8)
BMI (kg/m^2^)	23.6 (2.5)	23.9 (2.7)	23.8 (2.6)
Height (cm)	162.0 (6.4)	161.0 (7.6)	161.5 (7.0)
Weight (kg)	61.8 (6.1)	61.9 (7.1)	61.8 (6.6)

Changes in SARS-CoV-2 RT-PCR and CT value

By day 4, 85.4% of patients in the Imusil+SoC group tested negative for RT-PCR compared to 64% of patients exhibiting the same outcome in the SoC group (P=0.0156) (Table 3). The baseline CT value was comparable in both groups (Imusil+SoC, 27.8; SoC, 27.6; P=0.6859). After four days, no significant difference was observed in CT value in both group (P=0.1572) (Table 3).

**Table 3:** Changes in SARS-CoV-2 RT-PCR and CT value: ITT population (n=98) *Data shown as mean (SD) ITT, intention to treat; SoC, standard of care; CT, cycle threshold; SARS-CoV-2, severe acute respiratory syndrome coronavirus 2; RT-PCR, reverse transcription-polymerase chain reaction; SD, standard deviation

Visits	Imusil+SoC (n=48)	SoC (n=50)	P-value
SARS-CoV-2 positive RT-PCR, n (%)			
Screening/day 1	48 (100.0)	50 (100.0)	-
Day 4	7 (14.6)	18 (36.0)	0.0156
CT value*			
Screening/day 1	27.8 (2.6)	27.6 (2.7)	0.6859
Day 4	(n=7) 30.1 (0.7)	(n=18) 29.5 (1.6)	0.1572
Change in CT value	2.3	1.9	

Clinical improvement

After eight days, clinical improvement was observed in all patients from the Imusil+SoC group, while in the SoC group, clinical improvement was observed in 94.0% of patients (P=0.4947) (Table 4).

**Table 4:** Changes in clinical improvement: ITT population (n=98) ITT, intention to treat; SoC, standard of care

Visits	Imusil+SoC (n=48)	SoC (n=50)	P-value
Clinical improvement, n (%)			
Day 4	2 (4.2)	3 (6.0)	0.4000
Day 8	48 (100.0)	47 (94.0)	0.4947

Severity assessment

At baseline, most patients from the Imusil+SoC (95.8%) and SoC (98.0%) groups presented with mild COVID-19 symptoms (WHO COVID-19 ordinal scale score: 2). After four days, patients with symptom control (WHO COVID-19 ordinal scale score: 1) were significantly higher in the Imusil+SoC group (95.8%) than the SoC group (80.0%) (P=0.0411) (Table 5). After eight days, all patients from the Imusil+SoC group were in the WHO score of zero patients with no clinical symptoms, while 8.0% of patients from the SoC group still presented with mild COVID-19 symptoms (WHO COVID-19 ordinal scale score: 1) (P=0.1176) (Table 5).

**Table 5:** Summary of severity assessment of WHO ordinal scales: ITT population (n=98) Data shown as mean (SD) SoC, standard of care; SD, standard deviation; ITT, intention to treat; WHO, World Health Organization

Severity assessment	Baseline/day 1			Day 4			Day 8		
	Imusil+SoC (n=48)	SoC (n=50)	P-value	Imusil+SoC (n=48)	SoC (n=50)	P-value	Imusil+SoC (n=48)	SoC (n=50)	P-value
0: no clinical or virological evidence of infection	-	-	0.6134	1 (2.1)	3 (6.0)	0.0411	48 (100.0)	46 (92.0)	0.1176
1: no limitation of activities	-	-		46 (95.8)	40 (80.0)		-	4 (8.0)	
2: limitation of activities	46 (95.8)	49 (98.0)		1 (2.1)	7 (14.0)		-	-	
3: hospitalized and no oxygen therapy	2 (4.2)	1 (2.0)		-	-		-	-	

Change in inflammatory markers from baseline to day 8

**Figure 2:**
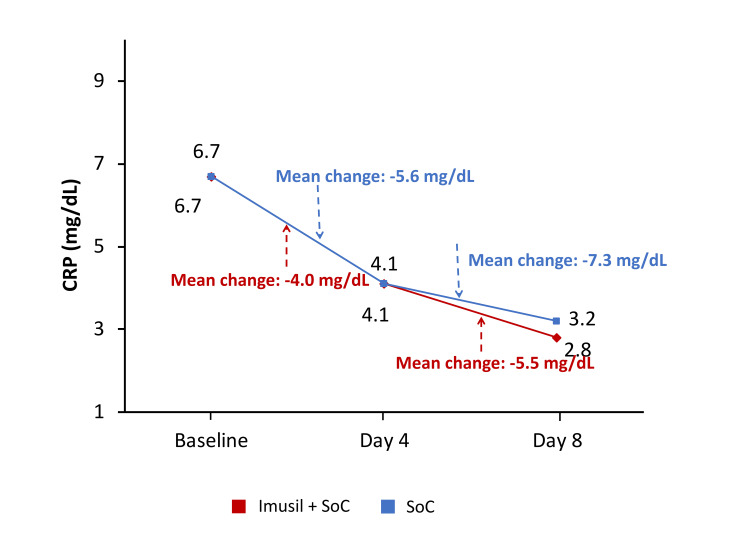
Change in CRP from baseline to day 8: ITT population (n=98) CRP, C-reactive protein; ITT, intention to treat; SoC, standard of care

**Figure 3:**
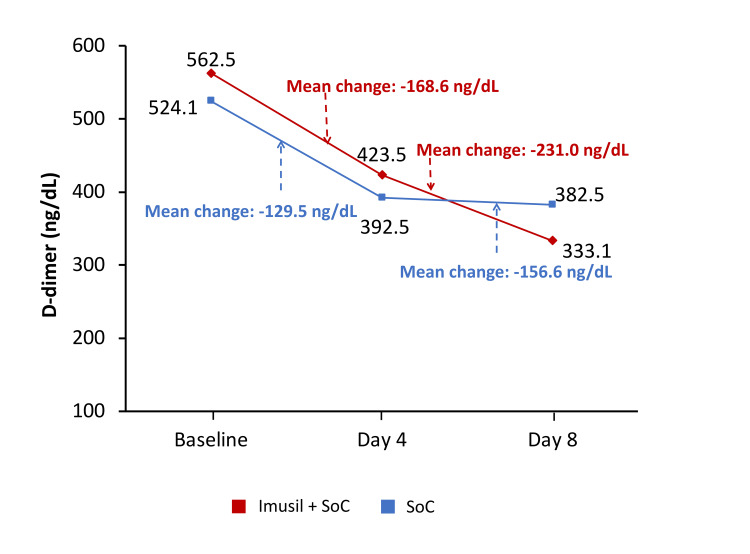
Change in D-dimer from baseline to day 8: ITT population (n=98) ITT, intention to treat; SoC, standard of care

**Figure 4:**
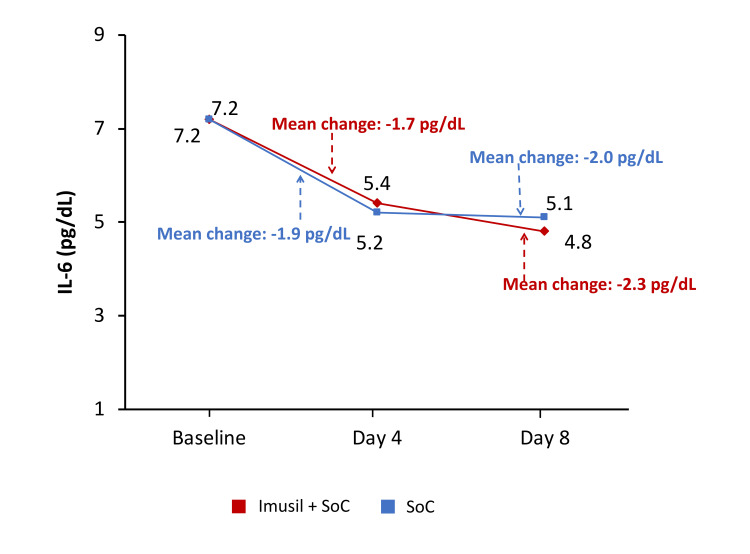
Change in IL-6 from baseline to day 8: ITT population (n=98) IL-6, interleukin 6; ITT, intention to treat; SoC, standard of care

Over the subsequent follow-up visits, the C-reactive protein (CRP) levels, level of D-dimer, and IL-6 levels decreased from baseline in both treatment groups, with no statistical difference between the groups at four days and at eight days (Figures 2-4). The decrease in the levels of CRP at eight days was comparatively higher in the Imusil+SoC group versus the SoC group (-7.3 mg/dL versus -5.5 mg/dL; P=0.2457). The decrease in the levels of D-dimer at eight days was also comparatively higher in the Imusil+SoC group versus the SoC group (-231.0 ng/mL versus -151.6 ng/mL; P=0.2689). The IL-6 levels had comparatively higher reduction with the Imusil+SoC group over the SoC group (-2.3 pg/mL versus -2.0 pg/mL; P=0.7685) at eight days.

Treatment-emergent AEs

There were no serious treatment-emergent AEs in the drug arm. Two patients from the placebo (SoC) arm reported vomiting (n=1) and pruritus (n=1).

Vital signs, physical examination, and laboratory evaluations

Patients from both groups reported normal pulse rate, respiratory rate, SpO_2_, temperature, and blood pressure during vital examinations. There was no statistical difference in the change in laboratory parameters between the Imusil+SoC and SoC groups (Table 6).

**Table 6:** Clinical laboratory evaluations: safety population (n=98) Data shown as median (range) SoC, standard of care; BUN, blood urea nitrogen; RBS, random blood sugar; SGOT, serum glutamic-oxaloacetic transaminase; SGPT, serum glutamate pyruvate transaminase; RBC, red blood cells; WBC, white blood cells

Parameters	Baseline/day 1		Day 8	
	Imusil+SoC (n=48)	SoC (n=50)	Imusil+SoC (n=48)	SoC (n=50)
Alkaline phosphatase (U/L)	115.0 (55.7-258.6)	107.3 (43.0-265.9)	100.7 (37.5-155.3)	96.4 (40.5-172.7)
BUN (mg/dL)	15.6 (7.6-56.9)	18.1 (7.9-42.5)	13.5 (8.0-68.5)	15.0 (8.2-65.5)
Basophils (10^9 ^/L)	0.0 (0.0-1.1)	0.1 (0.0-2.0)	0.2 (0.0-2.0)	0.3 (0.0-1.7)
Eosinophils (10^9^/L)	2.3 (0.1-11.1)	2.0 (0.0-9.8)	3.0 (0.0-12.9)	2.3 (0.0-12.9)
Hemoglobin (g/dL)	12.5 (8.5-17.0)	12.8 (8.5-17.4)	12.6 (9.7-16.0)	12.8 (8.9-16.8)
Lymphocytes (10^9^/L)	20.2 (5.0-40.0)	24.4 (10.0-49.0)	28.0 (10.9-45.0)	26.0 (13.1-56.7)
Monocytes (10^9^/L)	3.0 (1.0-9.2)	3.0 (1.0-10.0)	5.0 (1.0-10.3)	4.0 (1.0-15.4)
Neutrophils (10^9^/L)	70.8 (51.4-90.0)	70.0 (42.1-90.5)	65.0 (41.2-88.8)	68.3 (41.3-87.0)
Platelet count (lakhs/cumm)	2.2 (1.1-3.7)	2.3 (0.9-4.9)	2.3 (1.0-4.8)	2.2 (1.2-5.9)
RBS (mg/dL)	90.8 (57.3-147.6)	88.9 (52.0-165.1)	88.3 (40.0-142.0)	82.5 (40.0-139.4)
SGOT (U/L)	30.1 (10.2-49.2)	30.8 (9.1-57.0)	28.8 (13.3-69.5)	30.5 (10.3-89.9)
SGPT (U/L)	26.9 (7.2-59.2)	29.0 (5.4-65.6)	26.0 (6.8-59.1)	32.0 (6.0-58.4)
Serum bilirubin (mg/dL)	0.7 (0.1-1.6)	0.6 (0.2-1.2)	0.8 (0.1-1.5)	0.8 (0.2-1.7)
Serum creatinine (mg/dL)	0.9 (0.4-1.6)	0.9 (0.6-1.9)	0.9 (0.5-6.6)	0.9 (0.6-2.0)
RBC (10^9^/L)	4.4 (3.1-5.4)	4.5 (2.8-6.1)	4.5 (2.7-6.0)	4.6 (3.5-5.9)
WBC (10^9^/L)	7850.0 (3200.0-14730.0)	7780.0 (3000.0-13950.0)	7000.0 (2700.0-14360.0)	7430.0 (4100.0-19660.0)

## Discussion

Viral load in the respiratory tract and the duration of viral shedding are important determinants of COVID-19 transmission [14]. Viral load in the upper respiratory tract usually appears to peak around the time of symptom onset, and viral shedding begins approximately 2-3 days prior to the onset of symptoms [15]. Asymptomatic and pre-symptomatic COVID-19 patients can transmit COVID-19 infection [16-18]. Evidence indicates that viral load leading to systemic inflammation is one of the important predictors of morbidity and mortality in patients with COVID-19 [19]. Furthermore, viral load determines the duration of infectiousness; therefore, the early identification of infection and the timely use of treatment could benefit patients in achieving effective control [14].

Remdesivir, lopinavir/ritonavir, lopinavir/ritonavir with interferon beta-1α, and chloroquine or hydroxychloroquine are some of the chosen study drugs for the treatment of COVID-19 [20]. For patients with comorbidities, it is inevitable to take daily medication along with COVID-19 medications. Therefore, it is required to have safe pharmacotherapy for COVID-19 treatment along with antihypertensive, anti-asthmatic, and antidiabetic drugs. However, the abovementioned antiviral agents seem to have limited beneficial evidence in patients with comorbidities [7-9]. Thus, there is indeed a great rush to find the ayurvedic formulation against COVID-19.

Imusil is a proprietary ayurvedic medicine where the individual herbal ingredients have a variety of medicinal properties, including anti-inflammatory, antiviral, and immunomodulatory properties. The present study evaluated the antiviral efficacy and safety of a novel formulation Imusil, which is composed of kutki, guduchi, and amla.

The principal observation of the study is that RT-PCR negativity was faster and higher in the patients receiving Imusil. Patients in the Imusil+SoC group reported an improvement and recovery by 8+2 days of enrollment. However, comparatively lesser improvement was noted in the placebo arm.

It is interesting to note that a study by Patankar et al., which included 72 patients with mild and moderate COVID-19, showed significant clinical improvement in the symptoms post-polyherbal formulation treatment (4.3 versus. 1.7; P<0.0001) [21]. Narayanababu et al. showed that there was a clinical improvement in the mean ordinal scale from baseline to day 10 (WHO ordinal scale: 6.5-1.1) [22]. In concordance with Narayanababu et al.'s study, clinical improvement was observed in all patients post Imusil+SoC treatment [22].

Schulte et al. revealed that tumor necrosis factor-alpha (TNF-α) and IL-6 play a major role in the amplification of inflammatory response in COVID-19 infection [23]. Over the subsequent follow-up visits, the average CRP, D-dimer, and IL-6 levels decreased effectively in the Imusil+SoC group as compared to the SoC group. The previous study has shown a significant reduction in TNF-α and IL-6 levels post Imusil treatment [12]. In the previous study, an Imusil-treated cell showed significant reduction in TNF-α and IL-6 levels. Therefore, the results mentioned above, along with the present study, support the potent anti-inflammatory activity of Imusil.

Another randomized controlled trial using a polyherbal formulation and immune-boosting ingredients such as kutki has shown a significant reduction in CRP, D-dimer, and IL-6 levels from day 0 to day 14 [24]. Rastogi used the polyherbal formulation containing amla and also reported a significant change in CRP (baseline: 87.9 mg/L; post-treatment: 14.7 mg/L), D-dimer (baseline: 973.9 ng/mL; post-treatment: 225.0 ng/mL), and IL-6 (baseline: 149.4 pg/mL; post-treatment: 6.5 ng/mL) levels [25].

In this study, no serious treatment-emergent AEs were reported in the drug arm. In a systematic review and meta-analysis of 32 randomized controlled studies (n=3177), patients treated with herbal medicines demonstrated better clinical improvement with no adverse effects during the treatment period. None of the patients had SAEs requiring prolonged hospitalization or death (n=665; hazard ratio: 0.93; 95% CI: 0.76-1.14) [26]. Similarly, no drug-related AEs or SAEs were observed in patients utilizing the polyherbal AYUSH 64 formulation. None of the participants required invasive or non-invasive oxygen ventilation or developed complications such as acute respiratory distress syndrome, sepsis, or arrhythmia during the study period [27]. Thus, the results mentioned above, along with the present study, suggest that herbal interventions were relatively safe for the management of COVID-19.

Limitations

The pre- and post-analysis of clinical signs and symptoms within the group was not performed, which could have been valuable in understanding the impact on symptoms. Clinical trials with larger sample sizes and more extended follow-up periods can be conducted in the future. Lastly, another study on severely affected COVID-19 patients would be worthwhile to explore the efficacy and safety of Imusil among severe COVID-19 patients.

## Conclusions

Imusil, a polyherbal combination drug, provides effective antiviral activity in mild to moderate COVID-19 patients. Imusil ensures faster RT-PCR negativity, and it aids in the comparable reduction of markers such as CRP, D-dimer, and IL-6. It can be safely given along with SoC to enhance its therapeutic activity in patients with mild to moderate COVID-19 disease.
